# An impaired health related muscular fitness contributes to a reduced walking capacity in patients with schizophrenia: a cross-sectional study

**DOI:** 10.1186/1471-244X-13-5

**Published:** 2013-01-03

**Authors:** Davy Vancampfort, Michel Probst, Amber De Herdt, Rui Manuel Nunes Corredeira, Attilio Carraro, Dirk De Wachter, Marc De Hert

**Affiliations:** 1 University Psychiatric Centre Catholic University Leuven, Campus Kortenberg, Kortenberg, Belgium; 2 Faculty of Kinesiology and Rehabilitation Sciences, Catholic University Leuven, Leuven, Belgium; 3 CIAFEL - Centre for Research on Physical Activity, Health and Leisure, University of Porto, Porto, Portugal; 4 Department of Philosophy, Sociology, Education and Applied Psychology (FISPPA), University of Padua, Padova, Italy

**Keywords:** Muscle weakness, Fitness, Physical activity, Metabolic syndrome, Walking, Schizophrenia

## Abstract

**Background:**

Patients with schizophrenia report muscle weakness. The relation of this muscle weakness with performing daily life activities such as walking is however not yet studied. The aim of this study was to quantify walking capacity and health related muscular fitness in patients with schizophrenia compared with age**-**, gender and body mass index (BMI)**-**matched healthy controls. Secondly, we identified variables that could explain the variability in walking capacity and in health related muscular fitness in patients with schizophrenia.

**Methods:**

A total of 100 patients with schizophrenia and 40 healthy volunteers were initially screened. Eighty patients with schizophrenia (36.8±10.0 years) and the 40 age**-**, gender**-** and body mass index (BMI)**-**matched healthy volunteers (37.1±10.3 years) were finally included. All participants performed a standing broad jump test (SBJ) and a six-minute walk test (6MWT) and filled out the International Physical Activity Questionnaire. Patients additionally had a fasting metabolic laboratory screening and were assessed for psychiatric symptoms.

**Results:**

Patients with schizophrenia did have lower 6MWT (17.9%, p<0.001) [effect size (ES)=−1.01] and SBJ (14.1%, p<0.001) (ES=−0.57) scores. Patients were also less physically active (1291.0±1201.8 metabolic equivalent**-**minutes/week versus 2463.1±1365.3, p<0.001) (ES=−0.91) than controls. Schizophrenia patients with metabolic syndrome (MetS) (35%) had a 23.9% lower (p<0.001) SBJ**-**score and 22.4% (p<0.001) lower 6MWT**-**score than those without MetS. In multiple regression analysis, 71.8% of the variance in 6MWT was explained by muscular fitness, BMI, presence of MetS and physical activity participation, while 53.9% of the variance in SBJ-score was explained by age, illness duration, BMI and physical activity participation.

**Conclusions:**

The walking capacity and health**-**related muscular fitness are impaired in patients with schizophrenia and both should be a major focus in daily clinical practice and future research.

## Background

Patients with schizophrenia have a reduced aerobic capacity [1,2] and report subjective muscle weakness [3]. It is likely that both play an important role in the physical adaptation to daily life activities such as walking. Previous research already demonstrated that an impaired performance on daily life activities in patients with schizophrenia is associated with overweight, metabolic complications, smoking behaviour, negative symptoms and a lower physical self-perception [4-6]. An impaired functional walking capacity has been related as well to a reduced health related quality of life [7].

Recently, there has been a growing interest in the physical rehabilitation of patients with schizophrenia [8]. International guidelines state that physical activity should be one of the cornerstones in the multidisciplinary treatment [9,10]. It has been reported that physical activity might improve glycemic control, lowers blood pressure, improves lipid profile and decreases abdominal fat mass [11]. Unfortunately, only 25% of patients with schizophrenia meet the minimum health recommendations of 150 min of at least moderate intensity physical activity per week [12] and long**-**term adherence to supervised physical activity programmes is generally poor [11]. The failure to increase physical activity in patients with schizophrenia has been explained by socio**-**demographic, somatic and motivational factors including the presence of negative and depressive symptoms [13]. A prerequisite for supervised physical activity programmes in patients with schizophrenia therefore is information about the mental and physical health status including the presence of negative and depressive symptoms, the magnitude of impairment in aerobic capacity and functional exercise capacity and the metabolic and muscular fitness [14].

Although a reduced muscular fitness might contribute to daily life impairments, the extent of muscle weakness in schizophrenia patients compared with age**-**, gender and body mass index (BMI)**-**matched healthy subjects and its relation with performing daily life activities such as walking is, to the best of our knowledge, not yet studied.

The aim of this study therefore was to quantify walking capacity and health related muscular fitness in patients with schizophrenia compared with age**-**, gender and body mass index (BMI)**-**matched healthy controls. Secondly, we identified variables that could explain the variability in walking capacity and in health related muscular fitness in patients with schizophrenia.

## Methods

### Participants and procedure

Over a 6-month period, patients with schizophrenia of the University Psychiatric Centre of Kortenberg and the Brussels Nighthospital Belgium were invited to participate. Psychiatric diagnosis based on the Diagnostic and Statistical Manual of Mental Disorders, fourth edition (DSM**-**IV) criteria [15] was established by experienced psychiatrists responsible for the patients’ treatment. Patients were included if (a) acute symptoms were (at least partially) remitted, and (b) patients were stable on antipsychotic medication, i.e. using the same dosage for at least four weeks prior to inclusion. Patients were excluded if they had a DSM-IV diagnosis of substance dependence. Somatic exclusion criteria included evidence of significant cardiovascular, locomotor and endocrine disorders which, according to the American College of Sports Medicine [16], might prevent safe participation in the sub**-**maximal exercise tests. All participants received a physical examination and baseline electrocardiogram before testing.

Healthy control subjects were recruited among the personnel (i.e. health care workers, researchers, students) of the participating centres. All control subjects were volunteers who received a general physical examination in the previous year and reported to be free of significant psychiatric, cardiovascular, neuromuscular and endocrine disorders that might hinder safe participation. By selection, gender distribution and mean values for age and BMI did not differ significantly between healthy controls and schizophrenia patients. This matching was performed by an independent statistician blinded for the physical activity and physical fitness outcomes. All participants filled out a physical activity questionnaire and performed afterwards a muscular fitness and walk test. Participants were requested to refrain from eating, drinking coffee or smoking during a two-hour period prior to the tests. Patients were also screened for psychiatric symptoms and extrapyramidal side**-**effects of antipsychotic medication and received a fasting metabolic laboratory screening.

The study procedure was approved by the Scientific Committee of the University Psychiatric Centre of the Catholic University of Leuven, Belgium. All participants gave their written informed consent.

### Demographical data

Demographic patients’ data (including illness duration) were obtained from medical records while age for the control participants was self-reported. All participants were asked whether they currently smoked. Those participants who responded affirmatively to this question were asked how many cigarettes they smoked per day.

### Walking capacity

The six-minute walk test (6MWT) was performed according to the American Thoracic Society guidelines [17] in an indoor corridor with a minimum of external stimuli. Two cones, 25 m apart, indicated the length of the walkway. Participants were instructed to walk back and forth around the cones during six minutes, without running or jogging. Resting was allowed if necessary, but walking was to be resumed as soon as the participants were able to do so. The protocol stated that the testing was to be interrupted if threatening symptoms appeared. The total distance walked in 6 minutes was recorded to the nearest decimetre. Standardised encouragements were provided at recommended intervals. Supervision and measurement of the 6MWT was performed by one of four trained members (three physical therapists, one research nurse). With an intra class correlation of 0.96 [95% confidence interval (CI):0.94-0.98)], the 6MWT has been shown to be a reliable test to assess the exercise capacity in patients with schizophrenia [18].

### Health related muscular fitness

The health related muscular fitness was measured by a standing broad jump test [19], using a tape measure on a foam mat. Participants were asked to stand behind a line drawn perpendicular to the tape measure and jump forward as far as possible using arm swing and knee bending before jumping. The distance jumped was recorded from the take**-**off line to the farthest point backward of the participant. Higher scores indicate a better muscular fitness. The standing broad jump test previously showed an excellent reproducibility in patients with schizophrenia with an intra class correlation of 0.98 [95% confidence interval (CI):0.96-0.99)] [20].

### Physical activity participation

The International Physical Activity Questionnaire (IPAQ) [21] uses a structured format that asks participants to recall activities for each of the last seven preceding days in morning, afternoon, and evening time periods. Data from the IPAQ were summarised according to walking, moderate (activities that take moderate physical effort and make you breathe somewhat harder than normal such as carrying light loads, bicycling at a regular pace, or easy swimming), and vigorous activities (e.g., activities that take hard physical effort and make you breathe much harder than normal such as heavy lifting, digging, aerobics, or fast bicycling) per week. On the basis of what activities participants self**-**reported, the interviewer also clarified the perceived intensity of that specific activity. A continuous indicator was calculated as a sum of weekly metabolic equivalent (MET)**-**minutes per week of walking, moderate**-** and vigorous-intensity exercise. The MET energy expenditure was estimated by weighting the reported minutes per week within each activity category by a MET energy expenditure estimate assigned to each category of activity. MET**-**levels were obtained from Ainsworth et al. [22] The weighted MET**-**minutes per week were calculated as duration × frequency per week × MET**-**intensity, which were then summed across activity domains to produce a weighted estimate of total physical activity from all reported activities per week. Previous research indicated that the IPAQ can be considered as a reliable surveillance tool to assess levels of PA in patients with schizophrenia [12].

### Metabolic and anthropometric measurements

Body weight was measured in light clothing to the nearest 0.1 kg using a SECA beam balance scale, and height to the nearest 0.1 cm using a wall**-**mounted stadiometer. In patients with schizophrenia a 2**-**hour 75**-**g glucose load oral glucose tolerance test (OGTT) was performed according to previously proposed guidelines [23,24]. Patients were initiated on an overnight fast and were monitored during the OGTT. Waist circumference (WC) was measured to nearest 1 cm at the level of the umbilicus and at the end of expiration with the subject upright Blood pressure was recorded twice in the sitting position after a five minutes rest with an Omron M6 (HEM**-**7001**-**E) (Omron® Healthcare Europe). The average of both measures was taken. Patients received a full fasting laboratory screening. The presence of metabolic syndrome was assessed using the International Diabetes Federation criteria [25,26]. According to these criteria, for a patient to be defined as having the MetS they must have: central obesity (defined as waist circumference ≥94 cm for Europid men and ≥80 cm for Europid women, with ethnicity specific values for other groups) plus any two of the following four factors: (1) raised TG level: ≥150 mg/dL (1.7 mmol/L), or specific treatment for this lipid abnormality, (2) reduced HDL cholesterol: <40 mg/dL (1.03 mmol/L*) in males and <50 mg/dL (1.29 mmol/L*) in females, or specific treatment for this lipid abnormality, (3) raised blood pressure: systolic BP≥130 or diastolic BP≥85 mm Hg, or treatment of previously diagnosed hypertension, (4) raised fasting plasma glucose (FPG) ≥ 100 mg/dL (5.6 mmol/L), or previously diagnosed type 2 diabetes.

### Psychiatric and extra**-**pyramidal symptoms

Psychiatric and extra**-**pyramidal symptoms (EPS) were assessed by an independent and well trained nurse using the Psychosis Evaluation tool for Common use by Caregivers *(PECC) *[27]. The semi**-**structured PECC**-**interview evaluates 20 symptom items on a 7**-**point scale. Symptoms are grouped in 5 factors: positive, negative, depressive, cognitive and excitatory symptoms. The scores for each factor range from 4 to 28. The total score ranges from 20 to 140. EPS scores range from 4 to 16. Higher scores indicate more severe symptoms/side**-**effects. Validation results suggest that the PECC can be successfully used for the evaluation of these symptoms in schizophrenia [28].

### Medication use

Current antipsychotic medication use was recorded for each patient and converted into a daily equivalent dosage of chlorpromazine using Gardner's table [29].

### Statistical analyses

Unpaired t*-*tests with post hoc Bonferroni correction for continuous variables (p=0.005) and Fisher exact tests for categorical variables (gender) (p<0.01) were used to examine differences in characteristics between patients and healthy controls. Additionally, we calculated effect sizes (Cohen's *d*) to compare health related muscular fitness, walking capacity and physical activity participation between patients with schizophrenia and healthy controls. To identify independent predictors of the walking capacity and health related muscular fitness, a multiple stepwise regression analysis was carried out. Significance level was set here at p=0.01. Statistica 9 (Statsoft Inc, Tulsa, OK, 2009) was employed for the data analyses.

## Results

### Participants

A total of 100 patients with schizophrenia were initially screened. Six persons with co**-**morbid substance abuse were excluded while another six patients were excluded for a cardiovascular, locomotor or endocrine disorder that could prevent safe participation. Of the 88 eligible patients, another 8 declined to participate. Eighty participants were thus included in the final analysis. No significant differences in demographical and clinical variables were found between participants and drop**-**outs (data not presented). Forty healthy controls, matched for age, BMI and sex, agreed to participate. None of them dropped out. Participants’ characteristics are shown in Table 1. Sixty**-**five percent of the patients with schizophrenia smoked compared to 25% of the healthy controls. Smokers with schizophrenia consumed also more cigarettes per day. Except for the fact that in schizophrenia smokers were less physically active (MET**-**minutes per week: 984.1±853.2 versus 1830.8±1517.2, p<0.001), no statistical differences between smokers and non-smokers were found (data not presented). Mean duration of illness was 12.8±9.5 years. Mean daily equivalent dosage of chlorpromazine was 687.2±406.0 mg/day.

**Table 1:** Baseline characteristics of patients with schizophrenia and healthy controls

	**Schizophrenia**	**Healthy controls**	**p**
	**(n=80)**	**(n=40)**	
Gender (M/F)	55/25	27/13	0.64
Age (years)	36.8±10.0	37.1±10.3	0.88
BMI (kg/m^2^)	26.3±5.5	25.7±3.8	0.23
Smoking (cig/day)	14.8±14.8	3.8±6.7	<0.001*
PECC			
Positive symptoms	8.0±4.0	/	
Negative symptoms	10.4±4.5	/	
Cognitive symptoms	7.0±3.6	/	
Depressive symptoms	10.0±4.5	/	
Excitatory symptoms	7.2±3.3	/	
EPS	5.2±1.8	/	

Data expressed as mean ± standard deviation, BMI=body mass index, PECC= Psychosis evaluation tool for common use by caregivers, EPS= extra-pyramidal symptoms, *significant after Bonferroni-correction (p=0.005).

### Comparisons of walking capacity, health related muscular fitness, and physical activity participation between patients with schizophrenia and healthy controls

Patients with schizophrenia did have a significantly impaired walking capacity (17.9%) and health related muscular fitness (14.1%) compared to age**-**, gender**-** and BMI**-**matched healthy controls (Table 2). Except for the total minutes of walking per week, patients with schizophrenia were also significantly less physically active as measured with the IPAQ than age**-**, gender and BMI matched healthy controls (Table 2).

**Table 2:** Comparisons of health related muscular fitness, walking capacity and physical activity participation between patients with schizophrenia and healthy controls

	**Schizophrenia**	**Healthy controls**	***Δ***	**Effect size**	***p***
	**n=80**	**n=40**		**Cohen’s *d *[95%CI]**	
Standing broad jump (cm)	147.4±45.4	171.7±39.4	14.1%	−0.57 [−0.17 to −0.94]	0.003*
6MWT (m)	583.6±140.7	710.6±108.3	17.9%	−0.97 [−0.57 to −1.37]	<0.001*
IPAQ total minutes PA	326.9±306.7	563.2±293.9	41.9%	−0.79 [−0.39 to −1.17]	<0.001*
IPAQ total minutes of vigorous PA	36.4±81.7	88.9±88.4	59.0%	−0.62 [−0.24 to −1.01]	0.003*
IPAQ total minutes of moderate PA	110.0±181.7	298.8±263.3	63.2%	−0.89 [−0.49 to −1.29]	0.003*
IPAQ total minutes of walking	187.0±215.4	244.6±156.5	23.5%	−0.29 [0.09 to −0.67]	0.10
IPAQ total MET-minutes/week	1291.0±1201.8	2463.1±1365.3	47.6%	−0.93 [−0.53 to −1.33]	<0.001*

Data expressed as number (percentage) or mean ± standard deviation. Effect sizes were calculated using Cohen’s *d*. *Δ*=the difference in % between patients and controls, 6MWT= 6-minute walk test distance, IPAQ= International Physical Activity Questionnaire, PA=physical activity, MET= metabolic equivalent, *significant after Bonferroni-correction (p=0.005), CI=confidence interval.

### Correlations with walking capacity and health related muscular fitness in patients with schizophrenia

A longer distance on the standing broad jump test was significantly correlated with a longer distance achieved on the 6MWT (Table 3). Better performance on both physical fitness tests was significantly associated with lower BMI and higher physical activity levels, including total minutes of physical activity per week, total minutes of walking per week, total minutes of vigorous activity per week, the sum of weekly MET**-**minutes per week (Table 3). Younger age, a lower illness duration, smoking behaviour and less negative and depressive symptoms were only associated with a better health related muscular fitness, while a lower antipsychotic medication dose as expressed in chlorpromazine equivalents was associated with a higher 6MWT score (Table 3).

**Table 3:** Pearson correlations of health related muscular fitness and walking capacity with demographic and clinical characteristics in patients with schizophrenia

	**Standing broad jump**	**6MWT**
6MWT (m)	0.68**	/
Standing broad jump (cm)	/	0.68**
Age (years)	−0.31*	−0.28
Illness duration (years)	−0.53**	−0.20
BMI (kg/m^2^)	−0.46*	−0.61**
Cigarettes (cig/day)	−0.32**	−0.25
IPAQ total PA minutes°	0.45**	0.64**
IPAQ walking minutes°	0.37**	0.56**
IPAQ moderate PA minutes°	0.19	0.25
IPAQ vigorous PA minutes°	0.39**	0.37*
IPAQ MET-minutes/week	0.55**	0.69**
PECC Positive symptoms	0.20	0.17
PECC Negative symptoms	−0.33*	−0.29
PECC Depressive symptoms	−0.35*	−0.22
PECC Excitatory symptoms	−0.10	0.15
PECC Cognitive symptoms	−0.29	−0.18
Chlorpromazine equivalent	−0.19	−0.42**
EPS	−0.07	−0.23

*p<0.01, **p<0.001, °derived from the International Physical Activity Questionnaire, 6MWT=6-minute walk test distance, BMI=body mass index, IPAQ= International Physical Activity Questionnaire, PA=physical activity, MET= metabolic equivalent, PECC= Psychosis Evaluation tool for Common use by Caregivers, EPS=extrapyramidal side-effects.

### Differences in walking capacity and health related muscular fitness between patients with and without metabolic syndrome

Twenty**-**eight (35%) patients with schizophrenia did suffer from the metabolic syndrome. There were no significant differences in the presence of psychiatric symptoms or EPS between those with and without the metabolic syndrome. Sixteen patients with metabolic syndrome smoked (57.1%) compared with 24 (46.1%) in the group without metabolic syndrome. There was no difference in the number of cigarettes smoked per day between those with and without the metabolic syndrome (26.8±12.2 versus 21.0±12.5, p=0.14). In contrast, the distance achieved on the 6MWT was 22.4% less (491.4±75.3 m versus 633.2±92.1 m, p<0.001) and the health related muscular fitness 23.9% reduced (122.6±35.7 m versus 161.1±44.7, p<0.001) in patients with metabolic syndrome. Patients with metabolic syndrome walked also significantly less minutes per week than those without metabolic syndrome (89.8±87.9 min versus 239.4±244.5 min, p<0.001), while their weekly energy expenditure as expressed in MET**-**minutes per week (709.3±675.3 versus 1604.3±1307.7, p<0.001) was significantly lower.

### Determinants of the walking capacity in patients with schizophrenia

Significant correlates of the 6MWT and the presence or not of metabolic syndrome were included in a stepwise multiple regression analysis. For physical activity participation the overall MET**-**minutes/week was included. Muscular fitness, BMI, the presence of metabolic syndrome and physical activity participation were identified as independent predictors of the walking capacity. The model explained 71.8% of the variability in the distance achieved on the 6MWT (Table 4). Almost 46% of the variance in 6MWT could be explained by muscular fitness alone. An additional 10.2% was explained by BMI. MET**-**minutes/week and the presence of metabolic syndrome explained 11.0% and 4.7% more of the variance. Antipsychotic medication dose could not be identified as an independent predictor.

**Table 4:** Multiple stepwise regression analysis of the variables associated with the walking capacity score

	**Cumulative r**^**2**^	**p**	**SEE**
Standing broad jump	0.459	<0.001	81.2
Body mass index	0.561	<0.001	73.7
IPAQ MET-minutes/week	0.671	<0.001	64.1
Metabolic syndrome or not	0.718	<0.001	59.8

Criteria: probability of F-to-enter ≤0.5; probability of F-to-remove ≥0.10, r^2^= determination coefficient, SEE= standard error of estimate, IPAQ= International Physical Activity Questionnaire, MET= metabolic equivalent, *Significance level set at p<0.01.

### Determinants of health related muscular fitness in patients with schizophrenia

Except for the 6MWT score, all significant correlates for health related muscular fitness and the presence or not of metabolic syndrome were included in the stepwise multiple regression analysis (Table 5). For physical activity participation the overall MET**-**minutes/week was included. Age, illness duration, BMI and physical activity participation were identified as independent predictors of the health related muscular fitness. The model explained 53.9% of the variability in the standing broad jump score. Age explained 23.6% of the variance. An additional 7.3% was explained by illness duration. BMI and MET-minutes/week explained 12.4% and 10.6% more of the variance. The presence of metabolic syndrome, depressive and negative symptoms and smoking behaviour could not be identified as independent predictors.

**Table 5:** Multiple stepwise regression analysis of the variables associated with the health related muscular fitness score

	**Cumulative r**^**2**^	**p**	**SEE**
Age	0.226	<0.001*	40.3
Illness duration	0.291	<0.001*	38.6
Body mass index	0.433	<0.001*	35.2
IPAQ MET-minutes/week	0.539	<0.001*	31.9

Criteria: probability of F-to-enter ≤0.5; probability of F-to-remove ≥0.10, r^2^= determination coefficient, SEE= standard error of estimate, IPAQ= International Physical Activity Questionnaire, MET= metabolic equivalent, *Significance level set at p<0.01.

## Discussion

### General findings

The present study demonstrates that in patients with schizophrenia an impaired walking capacity compared to age**-**, gender**-** and BMI**-**matched healthy controls is associated with a reduced health related muscular fitness. Older age, a longer illness duration, a higher BMI and the current physical activity level were all identified as independent predictors of the health related muscular fitness.

Our data do offer more rigorous evidence for the previously reported association in patients with psychotic disorders between self**-**reported impairments in daily life functioning such as walking short distances and a reduced muscle strength (i.e. handgrip strength) [30]. The present study also adds to the current knowledge that schizophrenia patients with metabolic syndrome do have a decreased health related muscular fitness compared to those without metabolic syndrome. As was demonstrated in the regression analysis an increased BMI partially explains the variability in standing broad jump scores. Patients with metabolic syndrome had an increased BMI compared with patients without metabolic syndrome. However, since healthy controls were matched for BMI, BMI does not explain the differences in health related muscular fitness observed between patients and controls. Other illness**-**related mechanisms might contribute to the impaired muscular fitness. Our data demonstrate that patients with schizophrenia are at high risk for developing metabolic complications. Body fat, especially in the abdominal region, is a metabolically active tissue secreting catabolic (leptin, retinol**-**binding protein**-**4) agents [31]. The fact that next to age, illness duration additionally explains the variance in health related muscular fitness strengthens the hypothesis that illness**-**related factors other than the investigated psychiatric symptoms, might contribute to the impaired muscular fitness observed. However, the higher rates of cigarettes smoking in patients with schizophrenia might be a confounding variable. Although smoking behaviour could not be identified as independent predictor for health related muscular fitness in patients with schizophrenia, it cannot be excluded that differences in health related muscular fitness between patients and controls might only reflect differences in smoking behaviour.

We also did not find any association between health related muscular fitness, current antipsychotic medication use and EPS, which is in agreement with a previous study reporting that those on antipsychotic drugs exhibit the same neuromuscular pathology as drug**-**free patients [32]. This might indicate that neuromuscular changes are not related to the side**-**effects or the chronic course of antipsychotic medication use. Due to the limited sample size we were however not able to investigate associations of health related muscular fitness with specific antipsychotic agents.

### Clinical implications

The present findings indicate that patients with schizophrenia should improve their health related muscular fitness in order to maintain their walking capability. The current International Organization of Physical Therapy in Mental Health guidelines [33] do recommend that for substantial health benefits, patients with schizophrenia should not only do at a minimum 150 minutes a week of moderate-intensity, or 75 minutes of moderate-to-vigorous-intensity aerobic activity, but should also perform muscle-strengthening exercises that are of at least moderate intensity and involve all major muscle groups on two or more days a week [33,34].

### Limitations

The findings of the present study need to be interpreted with caution because of some methodological limitations. The limited sample size, especially of the healthy controls, and the reliance on self**-**reported physical activity, a method that is prone to both systematic and random errors [35], prevent us from making any firm conclusions. Nevertheless, there was a high response rate in the patients group which should prevent serious distortion of the results due to selection bias. Although in the healthy controls the IPAQ scores were in agreement with several other Belgian studies [36,37], the overall smoking prevalence rate was approximately 1.5 times lower than in the corresponding general population [38]. This way a selection bias in favour of healthier control volunteers cannot be excluded. Secondly, we also did not include parameters such as socio-economic status and educational level. Both parameters are known to be associated with physical fitness and the level of physical activity participation, also in patients with schizophrenia.[13]. Thirdly, because of the cross**-**sectional design of this study no direct inferences can be made about the causal relationship between the determinants found and the impaired walking capacity. However, the impaired walking capacity is most likely both the cause and the consequence of the determinants found. As indicated in the present study, there might be a negative spiral with physical inactivity leading to different metabolic complications, which in turn might affect both health related muscular fitness and physical activity participation. Fourthly, also the use of a single field-test to measure the health related muscular fitness should be considered as a limitation.

### Future research

Future studies should investigate potential neurobiological mechanisms for the decreased health related muscular fitness observed. Previous research [39] already found neuromuscular abnormalities either in the muscle biopsy or macro electromyographic investigations, in almost half of first**-**episode and chronic patients. It is however unclear to which extent these neuromuscular abnormalities, including atrophy of the muscle fibres, contribute to impairments in health related muscular fitness. In the same way it still needs to be explored whether metabolic syndrome induces peripheral neuropathy [40] and if its associated loss in peripheral sensation might be associated with impaired muscle strength. Secondly, more longitudinal research is needed on the effects of duration, side effects, and dosage of antipsychotic medication on the muscular fitness of patients with schizophrenia.

## Conclusions

The present study demonstrates that in patients with schizophrenia a reduced walking capacity is associated with an impaired health related muscular fitness. Older age, a longer illness duration, a higher BMI and the current physical activity level are all independent predictors of the health related muscular fitness. Both, the reduced walking capacity and impaired health related muscular fitness should be a major focus in daily clinical practice and future research.

## Competing interests

M. De Hert has been a consultant for, received grant/research support and honoraria from, and been on the speakers/advisory boards of AstraZeneca, Lundbeck JA, Janssen-Cilag, Eli Lilly, Pfizer, Sanofi**-**Aventis and Bristol**-**Myers Squibb. The other authors reports no financial relationships with commercial interests.

## Authors’ contributions

The study was designed by DV, MDH and MP. All data were collected by DV, MP and ADH. Statistical analyses were performed by AC and DV. DV, MDH, and MP wrote the first draft of the paper, and all other co-authors commented and contributed to the subsequent revisions. All authors have read and approved the final manuscript.

## Pre-publication history

The pre-publication history for this paper can be accessed here:

http://www.biomedcentral.com/1471-244X/13/5/prepub
